# Strongylus Armatus*Read before the 29th Annual Meeting of the United States Veterinary Medical Association at Boston, Sept. 21st, 1892.

**Published:** 1892-10

**Authors:** J. F. Winchester


### THE JOURNAL
OF
### COMPARATIVE MEDICINE AND VETERINARY ARCHIVES.

VOL. XIII. OCTOBER, 1892. NO. 10.

### STRONGYLUS ARMATUS.*

BY J. F. WINCHESTER, D. V. S.

### INTRODUCTORY REMARKS.

*Gentlemen*:—In presenting this subject to you I do not want to convey the idea that it is a new subject, or of recent origin. I simply desire that it may renew its youth, and to establish the fact that this condition does exist in other localities than the Continent. Judging from the literature in English on this subject, it has certainly been overlooked, for, with a few exceptions, only cases are reported that are interesting on account of their grossness. Owing to these facts it has been necessary for me to obtain the greater part of the literature from continental authors.

Finding that to be the case, I have had the able assistance of Dr. J. M. Parker, of Haverhill, in order that a part of the investigation might be original work. To him we are indebted for several original drawings, the enlarged plates from Neuman, mounted specimens, revised description of the parasite, beside several post mortem reports of horses that had lesions due to this nematode.

At this point it might be well to state that in man, according to Prof. W. F. Whitney, of Harvard Medical School, aneurisms have never been found due to a nematode.

### SYNONYMS.

*Armed Sclerostoma; Sclerostoma equinum; Palisade worm; Armed Strongyle.*

This parasite is a nematoid of the genera strongylus. The term "nematoid" derives its origin from the Greek word "nema,"

* Read before the 29th Annual Meeting of the United States Veterinary Medical Association at Boston, Sept. 21st, 1892.

signifying "a thread;" the term "strongylus" meaning round or cylindrical.

HISTORY.

A knowledge of these parasites goes back to the 17th century. In 1655 Ruysch discovered in an aneurism of the mesenteric artery of a horse an innumerable quantity of small worms, and later he published three or four similar observations. Schultze, in 1725; Chabert, in 1782, recorded similar instances, and since then such observations have been greatly multiplied, principally by Rudolphi, Hodgson, Greve, Rigot, and many others, including English and American veterinarians. Bollinger has made an attentive study of this parasitism, and in following it out has definitely established its essential points. These verminous aneurisms have only been found in the equidæ, and, according to Neuman, more frequently in the ass than in the horse. Hering asserted that, except in young foals, it is rare to find a horse without aneurismal dilatations, and Bollinger estimated that from 90 to 94 per cent. of adult horses are so affected. They are found principally in the large mesenteric artery and its branches, although sometimes found in the other arteries.

DESCRIPTION OF PARASITE.

Armed Sclerotoma.—"Body grey or brown, shaded with red; straight, rigid, the anterior part being broader than that which immediately follows." (See plate 1). "Mouth orbicular, widely open and rendered tense by several chitinous concentric rings, the innermost of which are garnished with fine teeth, while the outermost carry six papillæ symetrically divided." (On the outermost ring there is also a fringe of what appear like long, slender, sharp-pointed cilia; these are not seen when the mouth is closed; they are best shown by pressing on the cover glass, and so pressing the air out of the mouth, causing the cilia to spread outwards. The six papillæ mentioned by Neuman are sharp spicules, and are curved inwards, hook-like, and probably serve the purpose of attaching the worm to the mucous membrane).

"The buccal capsule is sustained by a longitudinal line or rib, and has at the bottom two round, sharp plates." (Opening out of the mouth is the pharyngeal capsule, which is armed anteriorly and is tunnel shaped; the posterior opening being the smaller. The anterior portion of the æsophagus is constricted and narrow for about a third of its length, when it begins to expand, forming the posterior ventricle or bulb).

"The caudal pouch of the male is almost trilobate, the posterior ribs being trifurcated, the middle double, the anterior cleft." (From the anterior portion of the pouch the spicula or male genital organ projects. It is first divided into two parts—deferens canals—which afterwards unite towards the extremity.) "The female, which is much thicker and larger than the male has the tail obtuse, and the vulva situated at the posterior moiety of the body," (and is surrounded by a roughened circular surface of a reddish brown color, which is grasped by the caudal pouch of the male during copulation. The uterus occupies a large part of the body; it can be made out very readily by the ova which are usually present in large numbers.) "The eggs are ovoid, and measure 92u long, 54u broad. The dimensions are variable; sometimes the males are 18 mm. to 20 mm. long, and the females 20 mm. to 26 mm. and at other times it is respectively 26 mm. to 35 mm. and 35 mm. to 55 mm. (The worms are found in an agamous or undeveloped state inthe blood vessels. (Plate II.) In this condition they are covered by a thin transparent membrane which entirely covers and encloses the still undeveloped worm. It has a temporary mouth and æsophagus, which lead directly into the still inperfectly formed mouth.)

This worm infests the cæcum and commencement of the large colon, and—with the Ascarius megacephala—is the worm most commonly found in the equidæ. The armed sclerostomes hold firmly by their buccal armature to the mucous membrane, which forms at the point of adherence a small dark prominence. They are frequently met with in couples—the two individuals forming an almost right angle, and adhering so intimately that they may be preserved in this condition in alcohol. Notwithstanding their sometimes considerable numbers, and the irritation they should produce in the mucous membrane, their presence in the horse is rarely betrayed by any appreciable symptom. They have sometimes been accused of causing death by anæmia, diarrhœa, colic, etc. It is not only on the internal surface of the large intestine that they are met with, for they are found in aneurisms of the mesenteric artery, and in the hepatic, renal, spermatic, occipital, and other arteries; in the muscles, pancreas (Goubaux, Montane), ligaments of the liver, and in submucous cysts of the cæcum, and sometimes of the duodenum. In all these instances they are in an agamous state, and represent one of the phases in the development of the species. With regard to the intestinal tumors, their volume varies from that of the head of a pin to that of a hazel or small almond-nut, according to the development attained by the worm inside each tumor. The latter also contains altered blood or pus, and there is more or less hyperæmia around the circumference. The worm within is rolled upon itself, and is, of course, of variable dimensions, sometimes extremely fine, and never so large as in the adult state; it is always destitute of reproductive organs. Occasionally there is no worm in the tumor, and then there is seen a small opening at the summit, by which it has escaped. The worms found in the organs mentioned above are also agamous; they represent the primary phase of development, as they do not become sexualized except in the cæcum and colon. Colin states that the armed sclerostomes are worms which migrate internally, and that their development is effected almost in one place. The ova are deposited in the substance of the intestinal mucous membrane—perhaps in the punctures produced by the mouth of the female, or perhaps merely in the orifices of the glands—and they are hatched there, the embryos becoming encysted at the points where they are hatched, in the cysts developed by their presence. After being developed and having undergone several moultings, they make their exit from the cyst, and fix themselves on the surface of the mucous membrane; though a certain number remain in their cyst, grow there, have the genital organs partly formed, but nevertheless always remain agamous. Those found in aneurisms and in the peri-intestinal organs, must have entered the blood vessels on their leaving the cyst, and in this way be carried—by a centrifugal migration—to the parts where they are found.

Baillet has shown that this is not the ordinary mode of reproduction and development of the sclerostomes. The ova are expelled along with the fæces, and become hatched in a few days if they are in a damp place. The embryos that issue from them are cylindroid, a third or a fourth of a millimetre long, somewhat obtuse in front, and have a filiform tail. If the conditions of humidity continue to be favorable, they gradually grow, their integument becomes folded and forms a kind of sheath in which the worm moults in an evident manner. Baillet has been able to keep them several months in this state, or after complete moulting. It is at this period that they enter the body of the horse in the water the animal drinks (or perhaps on green forage), undergo moulting if they have not already done so, and penetrate the substance of the mucous membrane. Leuckart asserts that the embryos should pass through an intermediate host before entering the intestine of the horse. But, however this may be, it is possible that after they have lodged themselves in the mucous membrane, a small number of embryos stay to fix themselves in the cysts which they cause to be formed. The majority reach the circulatory system, and install themselves in the abdominal arteries—principally at the origin of the great mesenteric; there they form aneurismal dilations, filled with a ragged clot that adheres to the inner surface of the vessel, and in this the helminths are located. These verminous aneurisms play an important part in the etiology of colics. After an indefinite sojourn in the aneurism, the worms leave it by allowing themselves to be carried by the blood, and in the course of time reach the cæcum, where they form the majority, if not the whole of the submucous cysts. Their last migration is, therefore, in reality, centripetal. After remaining an uncertain time in the tumor they had caused to form, and having grown, the sclerostomes forsake it, attach themselves to the mucous membrane, become sexualized and copulate.

An interesting observation of Railliet gives support to this theory as to the development of the sclerostomes. He found in a horse a considerable quantity of these worms in the cæcum, verminous cysts and walls of that viscus, and a smaller number in the duodenum and other parts of the small intestine.

The cysts in the duodenum—which are rare—were grouped on the small curvature of the intestine, and some were even observed disseminated in the mesentery. All of the latter contained sclerostomes still agamous ; but several of those in the small intestines—like those of the cæcum—had an opening in their centre and were vacant, the helminths having left them. This would seem to prove that the worms had reached the intestines by way of the arteries.

According to Semmer, at Dorpat, all the foals, without exception, have verminous aneurisms. Mather has witnessed a kind of epizooty break out among foals, consisting of verminous aneurisms of the aorta, near the origin of the renal arteries.

These aneurisms are only seen on certain visceral branches of the posterior aorta, and exceptionally on the posterior aorta itself.

In sixty-five horses, Hering has noticed aneurism of the trunk of the great mesenteric artery in seven cases ; the cœliac artery in fifty-nine cases ; the cæcal artery in eighteen cases ; the artery of the small intestine in sixteen cases ; the small mesenteric artery in two cases ; the cœliac trunk in two cases ; the hepatic artery in three cases ; and in the renal artery in one case. It is not uncommon to find more than one aneurism in the same horse.

In thirty-five horses Bollinger counted sixty aneurisms, and in adding these to the 108 seen by Hering in sixty-five horses, he reckoned that in 100 horses there were 168 aneurisms, of which 153 were in the large mesenteric artery and its branches, four in the cæliac trunk, three in the hepatic artery, three in the small mesenteric artery, three in the renal arteries, and two in the posterior aorta. In 100 horses, ninety to ninety-four had one or more verminous aneurisms. Sclerostomes were also several times found in the spermatic artery, and on three occasions in the cerebral arteries. Lastly, Roll indicates them as being found in the vena cava, and, according to Valentine, a specimen was discovered in the vena portæ at the Berne veterinary school.

The young form of the armed sclerostome—the aneurismal sclerostome—is found in various arteries in the horse, and those of the brain are not exempt. Three observations have been published. Albrecht reports the case of a horse which, during work, suddenly began to stagger; the eyes were fixed, and the respiration was noisy; there were remissions and relapses. Three hours after the first symptoms appeared, the head was carried low and inclined to the left, and there were convulsive moments of the neck and limbs. Soon it fell on the left side, became unconscious and manifested complete insensibility. In this state it was killed, and at the autopsy there were found diffuse meningitis, hæmorrhagic encephalitis, and in the middle lobe of the cerebellum a sclerostome which had probably arrived there when an embryo. Van Heill saw a three years old horse which was suddenly attacked with furious vertigo that lasted about a quarter of an hour. An autopsy revealed congestion of the brain and choroid plexus, while a free sclerostome was lodged in the cortical substance of the right hemisphere. Le Bihan found another worm of this kind in the occipital artery; rupture of the aneurism caused the death of the horse in less than ten minutes. Abildgaard discovered the filaria equina between the duramater and cranial arachnoid of a horse. The agamous form of this worm has been encountered more frequently in the spermatic than in the renal arteries of the horse. Gurlt had already noticed the presence of these worms in the vaginal sheath. Aitken once saw an armed sclerostome in the spermatic artery of a foal, and Baird found one in the testicle of a three-years-old horse; the gland was indurated and the envelopes infiltrated. Brodie published a similar case. At the London Veterinary College one worm was found in the spermatic artery of the ass, and another in a funiculitis consecutive to castration. It is remarkable the frequency with which these worms occur in the abnormal testicles of horses affected with abdominal cryptorchidism. We met with a case of this kind in May, 1883; and Simonin and Jacoulet encountered three in the space of two mohths, the testicles having undergone fibrous degeneration. On incising the testicle of a cryptorchid which was normal in structure, Degive met with an armed sclerostome. It would be interesting to ascertain the relation in frequency between cryptorchidism and testicular parasitism. What is certain, is that the concealed testicles of horses affected with abdominal cryptorchidism often exhibit such alterations as fibrous tumours or serous cysts.

In stallions affected with hydrocele, Schmidt and Pottinger have remarked one or two specimens of this worm in the vaginal sheath. Their presence is easily explained by the communication that exists between the abdominal cavity and that sheath.

### PATHOLOGICAL ANATOMY.

The verminous aneurism is usually fusiform, sometimes globular or cylindroid. Its average size is about that of a walnut; though it may not exceed that of a pea, or it may attain the dimensions of a man's head. It consists of a dilation of the affected artery, with hypertrophy of its walls. The dilation is sometimes absent, notwithstanding grave thrombic lesions in the vessel (Durieux). The external tunic is usually thickened, and variably indurated, according to the age of the tumor. It adheres firmly to the neighboring parts, and is more or less confounded with the surrounding connective tissues. The middle tunic is always hypertrophied, and sometimes very much so. Its thickness—which is ordinarily about a millimetre—may attain, and even exceed, two centimetres. At one time this thickening is due to simple hypertrophy of the tunic; at another time it is owing to inflammatory phenomena, with atrophy of the muscular fibres. The internal tunic is nearly always altered. It may present every degree of endarteritis, and of retrogressive metamorphosis—from partial thickenings, and a white milky tint, to ulceration, atheromatous transformation, and calcification; the latter, however, is always rare, and may exceptionally assume an aspect of real ossification. In the interior of the aneurism there is usually a fibrinous deposit—a thrombus—always adhering, though to a variable degree, to the internal membrane. It is more or less regular and consistent, and partially blocks the vessels; but there is always a canal in the middle for the passage of blood. This thrombus is often prolonged in the artery beyond the aneurism, both before and behind ; and its external layers are capable of becoming organized and undergoing softening. Its formation is essentially connected with the presence of the worms, the inflammatory processes—ulcerative and regressive—in the internal tunic, and the dilatation of the vessel. Decroly has published a remarkable case, in which the alteration in the aorta extended from the heart to the lumbar region. In the aneurism, worms are found in nine cases out of ten ; their average number is from nine to eleven, and varies between two and 121. When they are absent, the lesions have a chronic character ; but when they are present, then these are more or less acute. The parasites are young armed sclerostomes. They are rose-tinted, and their average length is from 1 to 3 cm. ; their sexual characters are already well defined, but their genital organs remain rudimentary. They undergo one moulting in this situation, in which their buccal armature assumes its definite characters. Rayer and Diesing, who erroneously considered them as a distinct variety, named them ; the former the strongylus armatus minor, and the latter the sclerostoma armatum aneurysmaticum.

Amongst the aneurismal sclerostomes, some are almost free in the cavity of the artery ; but the majority are more or less concealed in the layers of the thrombus—the head or tail usually projecting into the blood-stream. They are also found in the hypertrophied walls of the artery, in either the internal or middle coats, or between these two. Sometimes nothing is found of them except the integuments they left after their final moulting. The gravity of verminous aneurisms is due to the risk of rupture of the vessels, and more especially—as Bollinger has pointed out—to their influence on the frequency and seriousness of colics. The clot formed in the interior of the great mesenteric, or other arteries liable to these aneurisms, may throw off one or more fragments, which are carried by the blood, and constitute so many embolas in the arterial ramifications passing to the intestine. According to the size of the embolus, the obliterated artery is itself more or less voluminous, and the disturbance set up more or less serious ; there is sudden anæmia or ischæmia of that portion of the intestine to which the artery is distributed, and consequently paralysis of one or more of the sections of the digestive tube, the secretions and movements of which are suspended. Cohn and Panum have shown experimentally, in fact, that such are the results of embolism of the great mesenteric artery. The ischæmic portion of the intestine becomes at first pale, then of a dark red color ; the mucous membrane is swollen, there are hæmorrhagic infarcts, serous exudates, ecchymoses, and sometimes a considerable increase of the organ in volume. These phenomena—which are almost immediately consecutive—are related to the total absence of pressure in the capillaries of the artery, and even in the venous trunk succeeding them, as far as the next veinule, where the circulation can go on freely. The blood flows from this point towards the capillaries, where the tension is nearly nil, and soon causes engorgement, and even small hemorrhages. In consequence of all this disturbance, there are colics, which rapidly disappear if the obstructed artery is of small calibre; for the collaterals soon supplement it. The duration of these colics depends upon the facility with which this collateral circulation can be effected.

It is sometimes easier in a large branch nearer the trunk of emergence, and this explains why an attack of colic that appears very serious will quickly disappear. Otherwise, the establishment of the collateral circulation plays an important part in the post-ischæmic hyperæmia; and the collateral or compensatory hyperæmia is related to the increase of blood pressure in the vessels adjoining the obliterated one, as Feltz has shown. When it does not produce irreparable lesions, the equilibrium is quickly restored, and all trouble disappears.

The circulation disturbances of the intestine cause a local paralysis in it, stagnation and consequent fermentation of its contents, with an abundant production of gas. The enteralgia induced in the healthy portions causes energetic contractions, which frequently lead to volvulus and invaginations. Friedberger and Frohner have often observed the rotation on its axis of the left part of the large colon—that is, its free portion—which is, of all the intestinal divisions,the most liable to thromboses and embolisms Paralysis of the intestine often brings about rupture of it, the stomach, or the diaphragm, owing to fermentation and enormous accumulation of matter and gas therein.

In animals which have been cured of colic for some time, old lesions in the form of thromboses are often found in the branches of the great mesenteric artery, as well as in the corresponding veins, these vessels being partially or totally obliterated, and around them pigmentation of the peritoneum and other organs is usually observed. Bollinger says that on a square centimetre of surface, there are sometimes found five or six arterioles or veinules so obliterated.

At the autopsy of horses which have died from colic, it is often difficult to discover the obliterated artery and the seat of the embolus, because of the great development of the intestinal vessels, and more especially on account of their congested condition; so that much care and patience are needed in this search.

The effects of the aneurisms—the thromboses, and the embolisms—are evidently subordinate to their situation. The presence alone of the aneurism and its clot reduces the calibre of the great mesenteric artery, and consequently diminishes the supply of blood to the intestine; this is sufficient to explain the chronic indigestion troubles observed, and these effects are all the more marked if the diminution in the lumen is extended to a ramification, but they are especially so if the vessel becomes completely obstructed by a detached fragment of the thrombus. But as the arteries of the small intestine anastomose freely by inoscultation close to the concave curvature of the organ, embolism of one of the vessels is never a fatal accident. It is the same with obliteration of one of the two cæcal arteries; for the other which anastomoses with it near the point of the cæcum can assume its function, so that the attacks of colic pass off. But if the trunk of the right fasciculus of the great mesenteric artery is completely obstructed, the cæcum does not receive any blood, and death quickly ensues. The large colon receives its blood by the two colic arteries, which have an independent origin; so that it seldom happens that both are blocked at the same time. The floating colon would be exempt from the danger of aneurismal embolism but for its first artery, which is derived from the great mesenteric, the other arteries arise from the small mesenteric, in which aneurism is rare. In cases of death from aneurisms, the alterations described above are most frequently found. There may also be rupture of a verminous aneurism, and abdominal hæmorrhage. But, as Friedberger and Frohner have remarked, embolic colics may terminate in death in twelve to twenty-four hours, and before serious intestinal alterations have had time to occur. The testine is in such cases usually very distended by gas and obstructed. Verminous aneurisms rarely give rise to characteristic symptoms, and their presence is often only recognized when rupture has taken place, which rapidly terminates in death from internal hæmorrhage. On the occurrence of this accident—which coincides ordinarily with a severe effort—the animal crouches or sits on its hind-quarters, knuckles over at the fetlock joints, and falls as if struck with paraplegia; the pulse is thready, limbs cold, visible mucous membranes blanched, etc.; and, generally, the last moments of life are marked by signs of profound and violent suffering. Aneurisms of the aorta appear to be more liable to rupture than those of the great mesenteric artery. As a rule the blood flows into the peritoneal cavity, but sometimes rupture takes place directly into the intestinal canal. In eighteen cases collected by Bollinger, fifteen were of rupture into the abdominal cavity, and three into the intestine. The latter result might be recognized sometimes from the presence of blood which impregnates the fæces passed before death. Rupture of verminous aneurisms is attributable to the feeble resistance of their walls —which have lost their elasticity and contractility—and to the increased arterial pressure, resulting from diminution in the lumen of the vessel. Besides the cases of rapid death, there have been noted —as symptoms of aneurism of the posterior aorta—decrease in vigor of the animal, stiffness in movement of the hind-quarters, difficulty and pain in micturition, arching of the loins, infiltration and intermittent lameness of one or both hind-limbs, cramps and signs of paraplegia; but these indications are not sufficiently characteristic to afford a sure diagnosis, though they may arouse suspicions which will sometimes be confirmed by a rectal exploration.

Attacks of colic are the most frequent sign of verminous aneurism, and are the consequence of embolism in the branches of the diseased vessels; but neither are these symptoms characteristic. Sometimes the colic is sudden and acute and disappears in a short time, to reappear after a variable interval; it depends upon local obstruction, which is soon compensated for by neighboring anastomoses, and is usually ascribed to indigestion, as there is no appreciable cause. In other cases the colics are sub-acute and a little painful, and are due to sudden paralysis of a portion of the intestine; death soon follows; or the disease runs a chronic course, and is characterized by difficult digestion, constipation alterating with diarrhœa, slight colic, some fever, and a capricious appetite; it is a kind of intestinal catarrh, that may continue for some days, or even weeks, and terminates either in recovery, or which is more frequent, by marasmus, cachexia and death. Lastly, in some cases the embolic obstruction of the small arteries of the intestine—when often repeated—ends in hæmorrhagic enteritis, to which the animal succumbs in several days or weeks. Friedberger and Frohner attribute to this state the following symptoms: Diminution of appetite or complete inappetence, increased thirst and rare defaction; the fæcal pellets are small and dry at first, then become soft, pasty, and later, sanguinolent and fetid: the urine is acid, and rich in phosphates; the fever is intense and persistent, and the pulse small and quick; the general debility increases, the animals become emaciated and the abdomen retracted, and now and again there is coma. Frequently, after feeding there is general aggravation of the symptoms and colic. Death is often ushered in by febrile paroxysms, muscular tremors, shivering, coldness of the limbs, pallor of the mucous membranes, quickened, difficult, and rattling respiration, tumultuous beating of the heart, and considerable elevation of the rectal temperature.

To sum up, colics which have their origin in disturbance of the circulation, have no particular signs which would allow them to be distinguished with sufficient precision in the complex group of abdominal complaints.

During the series of years that I have been in practice, the subject and treatment of colic in horses has been most unsatisfactory to me, and the literature of our text books would not satisfactorily come to my aid. Why, I have often asked myself, do horses become subjects of colic? Why do they, as a rule, succumb after several attacks? Why do those attacks, as a rule, become more severe until the last, when they die? Why is it that the usual stereotyped colic drench or bolus will relieve, and then again it does not produce any impression whatever, apparently? Why is it that a case of colic recovers without any notice being taken of it?

Knowing that there is never an effect without a cause, I began to make my post-mortem examinations with more care, and open some of the leaves of the book of nature that I had been in the habit of passing over The result of this closer observation revealed to me the fact, that the vessels supplying the intestines with blood, were frequently abnormal and showed aneurisms. Opening these dilations I found parasites, nematodes, and, by referring to Cobbold, found that they were well known.

The question then presented itself to my mind—are we, as a body of practitioners, less observing than those gone before us? Do we look wise, give a decided opinion upon some everyday occurrence in practice, with the positiveness as probably has been done in colics, and know as little about the cause and lesions produced? Are we not, as a body of veterinarians, more inclined to open the book of charges than that of nature?

Do not think for a moment that I would in the least underrate the worth of, or honesty of, the members of this profession. I think the fault lies, if there is any, with the authors of our standard works. In this particular instance, the subject cannot be said to be spoken of at all ; and in one instance, the author, in referring to it, states that it does not occur in this country (England), although it appears quite frequently in Germany. In referring to the journals of the present day (in English) one can almost count the articles on this subject on his hand, and, that being the case, I came to the conclusion there must be a few veterinarians that are not aware of the importance of this subject.

The examinations and investigations I have made on this subject, with the able assistance of Dr. J. M. Parker, of Haverhill, only again very forcibly bring to my mind the fact, that we must not take too much for granted, but make original inquiry for ourselves. It is not necessary that all the work shall have to be done with the microscope. Is it not a fact, that in making post-mortem examinations we only look for a condition that, in our opinion, would cause death, and never once think enough about it to look, and try to find out if there is a tangible cause for the effect produced. How often have we made a post-mortem examination and been unable to find lesions that would satisfactorily explain the cause of death, when, possibly by a closer examination the cause might have shown itself ?

Certainly there must be cause for this laxity in our post-mortem examinations, and I am inclined to think that, perhaps, the principal cause may be laid at the door of our instructors in a great many instances, but oftentimes the pressure of business is the cause.

Can we afford to allow the coming generations of veterinarians to look back upon us as not having the same powers of observation as the generation gone before us ? Why is it that the continental literature is so much more instructive than the rest ? The articles on record regarding this malady are interesting, but, with the exception of Dr. W. L. Willams, they are confined to unusual cases where the parasites are very numerous, or the lesions gross.

In Dr. W. L. Williams' article the several cases he cites are in animals that had been in pasture and the season wet, thus favoring, according to the available life history of the S. armatus, the development of this worm. From my observation, it is not necessary that the animal should have been in pasture in order to harbor this worm, or at least, within a few years. I am of the opinion that in the larger majority of deaths from colic there will be found in the blood vessels evidence of, or the parasite itself. It is not to the exceptional cases that I desire to call your attention, but to those of everyday occurrence.

The following cases may be of interest at this point at giving direct evidence, in my opinion, that the worm in question does produce lesions that are a cause of embolism ; that the parasite is a cause of altered nutrition, and many cases the cause of death.

I will also cite several cases from the pen of Dr. Parker, which I think sustain the position taken on this subject.

*Brown Gelding*, 10 years, 1,000 pounds. History.—Same owner for two years ; never sick before with present owner.

Found the animal with abdominal pain, sharp and intermittent. Urine free, fæces scant. Looks around to the flank continually ; eructation of gas from stomach. Treatment would relieve for awhile, but after twelve hours sickness he died.

Post-mortem twelve hours after death. Bloated; dark-colored serum escapes from the abdomen when opened. The large intestines normal in color, but distended with gas. Small intestine black for a distance of about four feet, due to a twist. Rest of viscera normal. On opening the anterior mesenteric artery found a thickening of the intima, with a new growth ragged, in which were found worms.

*Brown Gelding*, 6 years, 1,400 pounds. History.—This animal has been subject to fainting spells during the last four months. He would fall down, lie a few minutes, get up apparently all right, and resume his work for several weeks or days.

This day he went to work apparently well but soon manifested pain, which gradually increased, with periods of ease, until noon, when death ensued. I found this animal bloated, in severe pain, pulse and respirations hurried and short. Treatment did not produce any apparent relief except for a short time.

Post-mortem four hours afterwards. Bloated ; serum escapes when abdomen is opened. The small intestines dark-blue ; the large, black. The veins of mesentery and intestines filled with blood. The mucous membrane of intestines black with blood, the sub-mucous membrane filled with straw-colored serum. The duodenum was contracted on itself for about eight inches, beginning at pyloric orifia. The thoracic cavity contained considerable clear serum, also, the pericardium which had spots of lymph on its surface. The endo-cardium, dark-colored, with apparently calcareous deposits in its substance and on the valves. The superficial blood vessels of the brain were filled with blood ; the third ventricle contained some light-colored serum and its plexus was hyperæmic. The anterior mesenteric artery was the seat of an aneurism, the walls being thickened, the intima showing a new ragged growth which nearly filled its space and was the seat of several nematoids.

*Grey Gelding*, 18 years, 1,450 pounds. History.—This horse has been subject to attacks of colic during the past three or four years, and would respond to the ordinary treatment readily. Killed on account of lost usefulness. Autopsy immediately after death.

Melanotic tumors under tail and along the posterior aorta. Apparently calcareous deposits on the valves of left heart ; otherwise, so far as I could see, was in a normal condition.

Dr J. W. Parker, of Haverhill, reports the following :

*Black Gelding*, 1,400 pounds. Was called at 3 P. M. Found horse thrashing and sweating profusely, and in great pain. Temperature 100 degrees F. Pulse weak and difficult to get, but about 80 to 90 per minute. Ears and limbs cold ; membranes pale ; eyes wild and anxious ; prognosis unfavorable ; applied hot packs and gave morphia. Temperature gradually rose to 102 degrees F., pulse more weak and rapid. He breathed hard and sighed occasionally and trembled, but did not attempt to lie down. These symptoms gradually grew worse until 7.30 P. M., when he died. Autopsy.—Body tympanitic ; abdominal cavity contained a quantity of serous fluid ; mesenteric blood vessels filled ; small intestines were, in places, almost black in color ; the serous membrane showed numerous hæmorrhagic spots ; the serous membrane of the colon in region of large blood vessels, showed exudation and extravasation of blood and serum ; the mucus membrane was easily torn and almost black in color, with an exudate of lymph in sub-mucus coat. Thorax and pericardial sac each contained considerable quantity of serum, but seemed otherwise normal. Aorta : The intima was of an even red rose color, and on opening the anterior mesenteric artery several warty excresences were found loosely adherent to the intima ; the walls were much thickened, and when cut open a large clot was exposed, and entangled in the clot were several nematoids. The intima of the artery above the clot was of an even red color, while, immediately below the clot, the membrane was seemingly normal in color.

*Black Gelding*, 16 years, 1,050 pounds. History.—For past two or three years, after having done extra work, he would leave his feed, and in course of a week or ten days would again be of service.

This A. M. showed symptoms of abdominal trouble, with acute pain, hurried respiration and quick pulse. He did not get any ease during his sickness and at times was almost uncontrollable. The duration of sickness was twenty-four hours. Post mortem twelve hours after death. Bloated, dark colored serum escapes from abdomen when opened, mesentery dark ; both the small and large intestines were dark in color and the mucous membrane of the same was very soft and dark. The contents of intestines very pungent. Kidney was soft ; liver, dry and hard. Thorax contained quantity of dark fluid ; mucous of heart, soft and easily torn. Intima of posterior aorta deep rose color. No evidence of strongylus armatus.

*Black Gelding*, 12 years, 1,150 pounds. History.—Been subject to colic for some time. Called at 4 A. M. Found him bloated, cold sweat, mucous membranes pale, pulse fast and hard, respirations quick and labored. Died at 9 A. M.

Autopsy at 4 P. M. Bloated ; serous coat of intestines dark blue and blood vessels of mesentery and intestines filled with blood. Mucous membrane of large intestine apoplectic, with exudation of serum into the sub-mucous membrane. Rest of viscera apparently normal. Found aneurism of anterior mesenteric artery; walls thickened, the intima showed a new formation, ragged in appearance, which harbored several worms, and dark colored clots of blood were found below the thrombus.

*Black Gelding*, 12–14 years, 1,100 pounds. History.—Sick all day, not much pain, uneasy. At 8 P. M. Pulse 36. Respiration 18. Slightly bloated, easy, passes flatus. No fæces since A. M. Gave usual treatment and went away. 4 A. M., night man was awakened, went to the horse ; died soon after. Post mortem 11 A. M. same day.

Bloated ; removing abdominal muscles, large quantity of dark colored serum escaped ; indigested food around liver and stomach and small intestines. Stomach with small rupture, the edges rough, having clotted blood under external coat of stomach. Small intestines were blushed and contained a quantity of light colored mucus, sweetish to smell. The M. M. B. of large intestines, colon and cæcum, was apoplectic and fæces fluid. Liver soft and friable. Rest of viscera apparently normal. The internal lining of anterior mesenteric artery thickened and presented a cauliflower look, covering a space of about a quarter dollar, which had in its meshes several parasites. Growths were found on bicuspid valves, hard and glistening.

*Brown Gelding*, 9 years, 1,100 pounds. Taken sick 1 A. M. Saw him 3 A. M, and found him bloated, dull pain, up and down. Pulse small, hard and seventy-two. Respiration labored and short, cold sweat, visible M. M. B. pale. Usual treatment with relief for a while, but gradually failed until death ensued at 8 A.M.

Post mortem at 4 P. M., same day. Visible M. M. B. pale, bloated, mucus discharge from nose and mouth. Opened abdomen, found peritonitis. The colon serous coat, dark blue ; mucus coat, apoplectic. Small guts, serous coat dark in color; mucus M. B., normal. Rest of viscera apparently normal. Left side of heart contained a straw-colored clot. Anterior mesenteric artery aneurismal, containing a cauliflower growth, with several small parasites, and beyond this found a firm dark-colored clot filling artery.

This horse was taken sick with colic the 5th, recovered; went to work the 6th, but had to be returned in a few hours; the 9th he again resumed his work, and continued apparently all right until the A. M. of the 12th, when he died as above stated.

*Black Gelding*, 5 years, 1,050 pounds. Saw case at 6 P. M. Pulse full and strong; respiration little hurried; urinates freely; fæces plenty; restless, up and down. Prescribed for him, which gave ease for a while. 9 P. M., bloated; gave drench, slacked away and remained easy for a while, but continued in dull pain. 1 A. M., breathing hard and fast; sweating profusely; again relieved him and he remained comfortable until about 10 A. M., when I repeated the treatment which rendered him comfortable until 4 P. M., when he had a collapse without bloating; again did my best which caused him to remain quiet until 4 A. M. of the next day, when he threw himself down, pounding his head and striking with his fore feet, while the hind legs remained very stiff until a short time before death, which took place at 7 A. M. the same day. Post mortem, 30 hours after death, bloated. The abdomidal viscera, with the exception of about eighteen inches of small colon, were apparently normal. That part of the gut was inflamed and the contents dry. The M. M. B. of cæcum was well covered with dark raised spots and contained quite a number of parasites. One of the branches of the anterior mesenteric artery contained a growth on its internal surface which nearly filled its space, and in and around this were found parasites quite active.

*Brown Gelding*, 6 years, 1,400 pounds. Lawrence Ice Co. Taken sick about 5 P. M. Saw him at 6 P. M. Found discharge of indigested food from both nostrils ; anxious expression ; pulse full, about 80 ; M. M. B. of eyes and mouth pale ; breathing short ; cold sweat; no bloating to be seen. 8 P. M., bloated, hurried and short breath, pulse imperceptible, cold, M. M. B. of eyes still pale, that of mouth regained color, stands up braced ; tapped, no relief given ; died at 9 P. M.

Autopsy 15 at 11 A. M. Bloated, part of small colon protruding from anus ; opened abdomen, escape of bloody serum and indigested food. Vessels of mesentery full of blood ; those of small intestine engorged ; colon and cæcum apparently normal, as well as kidney, spleen and liver. Stomach ruptured, with blood and serum under torn edges. The stomach contained quite a number of bots. Small intestines, except part of duodenum, full of bloody serum ; large intestines contents semi-fluid. Cæcum contained quite a number of parasites S. Armatus. Rest of viscera apparently normal. The posterior aorta rose color inside, and clots of blood where anterior mesenteric artery given off. Anterior mesenteric artery contained a ragged new growth with walls thickened, but did not find a parasite.

Report of cases from the note book of Dr. J. W. Parker. Was called June 6, to see a case of colic, and found him presenting the usual symptoms and no history of having before had colic. He was under treatment for eight days, with days of ease, and was apparently convalescing, but death ensued the 15th inst.

Autopsy, following day. No bloating ; opening the abdominal cavity there was good deal of post mortem discoloration. Bowels were full of watery fæces ; mucous membrane was excoriated and much inflamed ; diaphragmatic flexture of colon was adherent to abdominal wall, where there was a patch of intense red discoloration, and in the centre was an abscess, opening into the walls of the colon, and containing two or three ounces of creamy pus. On examining the aorta and anterior mesenteric artery there was a clot and aneurism of the colic branch of the anterior mesenteric. The colic artery was full of dark colored blood, and about eight inches down a nematode was found (Filaria papillosa) ; immediately below this there was a long, slender straw-colored ante-mortem clot.

Dr. C. W. Stiles reports on the Filaria papillosa as follows : The parasite is the F. equinæ (papillosa) female. As a rule the nematode is found in the body cavity, but it is quite frequently found in the peritoneal cavity, more rarely in the pleural cavity of the horse, mule and ass. Generally, only a few specimens are found in one host, occasionally, however, a larger number are found. The females are much more frequent than the male. The same parasite has also been reported from the arachnoid, sub-peritoneal connective tissue and diaphragm, and the nematodes found in the eye are thought to be embryos of this species. Barnchello, also, believes that the nematodes found in the cutaneous helminthiosis of the horse also belong to this species.

*Roan Mare*, not subject to colic. Taken on the road, was very violent and lived about four hours.

Autopsy, 48 hours after. Portions of small intestines almost black in color; blood vessels filled. Walls anterior mesenteric artery thickened and roughened with adherent blood clot, with aneurismal dilation about size of hen's eggs, and entangled in the clot there were several nematodes (S. armatus).

*Kavanagh Mare*, taken suddenly sick about 2 P. M. (Flatulent colic). Chloral Hydrate and Arom. Spt. of Ammon. Later used turpentine and linseed oil and carbolic acid, with rectal injections of linseed oil and trocar and camila. Diagnosed twist of bowels. She died about 7.30.

Autopsy, held next day. Showed abdominal viscera fully distended and tympanitic, not much inflammation, no peritonitis. In one place on intestines there was a small patch of hæmorrhagic inflammation. The gastro spleenic omentum was intensely inflamed and dark in color. Thoracic viscera. Lungs slightly congested. Heart distended full. On opening intestines they were found to contain quantity of liquid fæces. Stomach was distended and contained gas and fermented food. Spleen was enormously distended; capsule tense, almost to bursting. This distention was especially well marked at the apex, where it was full to bursting. It was here that the gastro spleenic omentum was dark-red in color and intensely injected. On opening the posterior aorta the cæliac axis contained a quantity of clotted blood, and in one of the branches the intima was thickened and roughened in places, and adhering to, and partially concealed by, the roughened intima there were several nematodes. The anterior and posterior mesenteric arteries were normal.

On August 12th I was sent for to see a mare, which I found suffering a good deal of pain and presenting all the symptoms of acute lymphangitis. Temperature, 101½, pulse hard and full, and about sixty. Gave linseed oil with belladonna and veratrine inside, and ordered continuous bathing with hot vinegar, and water, and soap, and camphor liniment. The next day I got word that she was better; was eating better and seemed brighter, and the swelling was much reduced. The same evening I got word to come out at once as the mare was down, unable to get up, and was thrashing badly. I found her lying on her sore leg trying to get up, and becoming almost frantic in her endeavors. I at once turned her over on her other side, when she got up with little difficulty and at once began eating hay, and whinnied when water was brought. The following morning, much to my surprise, I received word that she was dead, and I immediately went out to make an autopsy.

Autopsy.—I found body tympanitic, swelling in leg much reduced; owner said she died easily, without struggling; so much so, that although he was in the same barn, he did not know she was dead till he went in to look at her. On opening abdominal cavity there were no lesions to be observed in the abdominal viscera, except one small patch of redness, with a few hæmorrhagic spots at the pelvic flexure of the colon. Thorax: The lungs were dark-colored and congested. The heart full on both sides. In the anterior mesenteric artery there were two aneurismal dilations, and in them there were several specimens of the strongylus armatus. In the sub-mucous coat of the cæcum there were several well-marked prominences, and through the thin membrane the coiled-up worm could be seen distinctly. Through want of tools and time I did not take out the brain.

Horse belonging to American Express Co., aged, died suddenly on the street while working (the day was cool and cloudy.) The horse fell down. "all in a heap," with his legs under him. It got up again, but it immediately fell, and died without a struggle. Autopsy. Held next day, eighteen hours after death. Body tympanitic, abdominal organs normal, no parasites in intestines. Thoracic organs. Lungs congested; heart empty; muscles dark. Aorta, normal. Kidneys, normal. Brain: On the base of the brain, around the pons there was a quantity of congestion and effusion. In the third ventricle there was a teaspoonful of serous effusion. The blood vessels in and around the vermiform process and the lateral lobes of the cerebellum were intensely congested. No strongylus were discovered.

POST MORTEMS OF HORSES FOUND AT THE KNACKER.

*Brown Gelding*. Aged. Killed. S. armatus abundant in cæcum; aneurism anterior mesenteric artery, with worms.

*Gray Mare*. Overdrove; died of exhaustion. No evidence of S. armatus in cæcum; small aneurism anterior mesenteric artery, but no evidence of parisite there. Owner unknown. Died colic. Apoplexy large intestines. S. armatus plenty in cæcum; anterior mesenteric artery aneurismal, with worms.

*Bay Gelding*. Old owner unknown. Died colic. Aneurism post aorta, at point where anterior mesenteric artery goes off, size of large pear, involving anterior mesenteric artery with thrombus and strongylus present, and one of diverging arteries completely plugged. No. S. armatus found in cæcum.

*Bay Gelding*. Killed for lost usefulness. Strongylus in cæcum;

Aneurism anterior mesenteric artery, with thrombus and strongylus.

*Gray Gelding.* Died from effects of heat. No evidence of parasite in question.

Mr. Mather, M.R.C.V.S., in *Veterinarian* for 1857, Vol. XXX, Page 190, writes as follows: "About twelve months since, when practicing in the South, the following cases came under my notice, and never having read in any of our veterinary works, or heard mention made of such a disease (excepting that veterinary surgeons had occasionally met with it), I thought perhaps you might deem the following particulars not unworthy of a place in the *Veterinarian.* It was thus only by chance that I was enabled to learn the nature of the complaint the animals were laboring under, and that in the following manner. The subjects of the disease were blood foals, varying in age from seven to sixteen months, and one of them having been found dead in the field, I was sent for to make a post mortem examination, it being suspected that the animals had been poisoned. On examining the foal previously to opening it, I found the body to be very much emaciated, and that the abdomen was greatly enlarged; on percussion of the belly, I detected the presence of a small quantity of gas, mingled with a fluid which I concluded was of a serous nature. From this circumstance I came to the conclusion that the animal had died from ascites, but, on opening it, I found the abdomen to contain quite a gallon of pure blood. On removing the viscera, I at once saw that the hæmhorrhage had come from a rupture of the posterior aorta, just in front of the renal arteries. I dissected out the vessel to nearly its whole length, and on examining it, I thought at first that simple aneurism existed; but, on cutting into the dilated portion near to the rupture, I found, much to my surprise, that the vessel was completely choked with myriads of small worms, similar in appearance to the filaria which we find in the bronchial tubes of calves suffering from bronchitis or husk. The internal coat of the vessel was considerably thickened; in fact, it appeared to be lined with a false membrane, which, no doubt, had been caused by the irritation set up by these creatures; in all the arteries given off from the main trunk were more or less of these parasites. About a fortnight from the time of being called to this case, I was sent for to see another of these foals, which, the man informed me, had been found down and unable to rise. On examining it, I observed that the pulse at the jaw was nearly imperceptible, the mucous membranes blanched, and the body very cold. I informed the owner that I was sure the foal was dying from internal hæmorrhage, and that, in my opinion, it was suffering from the same complaint as the last. We managed, however, after some difficulty to get the animal on his legs again; and immediately we had done so, it commenced voiding a large quantity of blood from the penis. Seeing that there was no chance of recovery, we had the foal destroyed. When I made my examination I found the bladder distended to pepletion with blood. The right kidney was twice its normal size, and in cutting into it I found it filled with similar parasites. The renal artery was quite as large as one's finger, and it, also, contained a large number of these creatures. The posterior aorta contained thousands of them.

Two other foals which were on the premises, I felt sure from their appearance were laboring under the same complaint, and the owner wished me to try if I could do anything for them. I must here say, that these foals had been taken off the mares at about six months old and placed on some cold, wet lands, where they had remained up to the time of my seeing them. The two surviving ones I had taken up and put into the bay of a barn, so that they could be supplied with crushed oats, pea meal, and good hay. I first gave them for three successive mornings Ol Tereb. two drachms, Ol. Lini. two ounces, and afterwards administered for a few days the following tonic—Ferri Sulph. half drachm, Pulv. Gent. one drachm. These remedies were alternately employed for a fortnight and I considered that the animals were improving under their use, but at this time the owner saw fit to dispose of the foals, so that I afterwards lost sight of them entirely. (Editor. Mr. Mather will find in the records of veterinary medicine several analgous cases to those he has related. They are not uncommon, and have frequently been brought before the notice of the students of the college by the professors.)"

Dr. W. L. Williams in his article says: "How often these parasitic thrombi occur amongst our horses we do not attempt to say; we have only looked for strongyles where they have been expected and have not been disappointed in any case. A positive diagnosis is generally available, by manual exploration per rectum, or by pulse, and respirations are more uniformly accelerated than in colic, and there is a peculiar, anxious expression about the animal that bodes no good, and leads one to conclude that he is dealing neither with ordinary colic nor enteritis "

I think Dr. Williams is right when he says we do not know how often these parasitic thrombi occur amongst our horses, for the reason there has never been enough careful post mortems to answer the question. I am not suprised that he finds them so readily when looking for them, for, he says—where expected they were always found. I must differ with him when he says a positive diagnosis is generally available, by manual exploration per rectum or pulse, respiration, and general expression of the animal. I think that in most cases the diagnosis follows death, although one is justified in expecting the presence of the parasite in the majority of cases that die from colic.

Dr. A. W. Clement in *Journal of Comparative Medicine and Surgery*, Vol. XIII, page 186, cites a very interesting case of rupture of the anterior mesenteric artery, but here the unusual case presents itself.

Dr. J. T. Duncan, in *Veterinary Journal*, March, 1887, cites several interesting cases that do not correspond to the every day occurrence of the practitioner, although the lesions produced were due to S. armatus.

Dr. R. W. Burke, in *Journal of Comparative Medicine and Surgery*, Vol. XIII, has an article on this subject, but does not cite any cases that have come under his observation.

Dr. C. A. Cary, in *Journal of Comparative Medicine and Surgery*, June, 1892, cites two post mortems of colts that never had colic; one died from tetanus, the other was killed on account of malformation; with aneurisms of anterior mesenteric in both, and living sclerostomes. Using these cases for an analogy, he thinks that the theory of verminous aneurisms causing colic should be more carefully looked into.

Mr. M. Laquerriere, in his article on colic, *Veterinary Review*, Vol. IX, page 222, says: "As to the predisposition produced by a diseased condition of the great mesenteric artery (aneurism) upon which Zundel places so much weight, we attach but little importance to it; but, on the contrary, we recognize the predisposition accompanying advanced age."

Prof. F. Smith, Army Veterinary School, Aldershot, has a very interesting article in *Veterinary Journal* for July, 1892, on "Intestinal Obstruction in the Horse," and I fail to find that he made mention of Strongylus Armatus as a cause for the same. He does say that "The rapidity of death depends entirely upon whether the blood supply to the bowel is partly or completely cut off, viz., a partial or complete twist." I am of the opinion that the twist to which he attaches so much importance is a result due to enteralgia, and that due to altered nutrition of the part. It is a fact that this parasite is not a stranger, but is readily found if looked for, and is a cause of thrombosis and embolism.

Mr. Hunting, in discussing Prof. Smith's paper, held "that the impacted food was the cause of the spasm or the pain, and that the proper thing was to remove the cause, even if they inflicted a little more pain." Does not the establishment of collateral circulation to the afflicted parts cause a return of normal state, and pain then cease, allowing that it is due to an embolism? Who can make a positive diagnosis of this condition during life?

Mr. H. G. Rogers said he was rather skeptical as to the condition called "twist," for the report usually came from Knacker in abdominal cases "Twist, Sir," and this is similar to the report from this side of ruptured diaphragm. He thought that sluggish liver and defective teeth were the causes of intestinal obstruction.

Prof. Macqueen thought there was another form of obstruction which demanded a little more attention than it usually received, and that was the form of obstruction due to aneurism of the branches of the mesenteric artery. He suggested that more careful post mortems might reveal a cause that is often overlooked.

Dr. Wm. Willis, M. R. C. V. S., in *Veterinary Record*, July 16, 1892, in criticising Prof. Smith's paper, remarks the entire absence of any reference to the condition of the circulatory apparatus of the bowels. He says, "I am inclined to think mesenteric aneurism is much more frequently responsible for the death of our patients than is generally supposed. Certainly it is not the rare disease of old horses, which the scant notices of our text books might lead one to believe. It is a most common condition. Physiological experiments teach us that when the blood supply to the bowel is suddenly interrupted, violent intestinal movements result, and that, if the interruption be continued, a paralytic condition of bowel results. I would suggest that the first result is probably the precursor of twist, and the second is certainly the explanation of some cases of obstructed colon."

How far mesenteric aneurism, thrombosis and embolism, are likely to give rise to the conditions referred to above, and to what extent they must be held responsible for the fatal termination of many of our bowel cases, is a matter which deserves more attention at the hands of the British veterinarians than it has hitherto received.

Dr. Willis cites seven post mortems: Five eight years old and under, two more aged. He found six with aneurisms: one of them with strongyles. The other one revealed a perfectly round hole, about the size of a sixpence, in the side of ileum, near ileo-cæcal valve. I am of the opinion, that, had his examinations been made with a little more care, he would have found the worm in each case, although not necessarily at the point of aneurism.

J. E. Miller, M. R. C. V. S., *Veterinary Record*, June 18, 1892, cites an exceptional case of mesenteric tumor with aneurism of anterior mesenteric artery.

Cobbold says, "So practically important, however, do I deem Bollinger's summary of the whole subject in relation to the hippopathological aspect of parasitism, that I feel it desirable to record his conclusions at full length. No professional man having any pretensions to a knowledge of the veterinary art—or, for that matter, to parasitism in relation to sanitation—should remain uninformed on this subject. Dr. Bollinger's results are thus stated :

1. The worm aneurisms of the visceral arteries of the horse, existing in 90 to 94 per cent. of adult horses, has a general correspondence with the aneurisma verum mixtum of man. It is, however, distinguishable from the same by its seat, cause, character of its walls, contents, and mode of termination. The worm-aneurism arises from a parasitism of the palisade worm (Strongylus armatus), owing to an inflammatory affection of the arterial walls which it causes, and which one may describe as a recurrent traumatic endoarteritis. This holds good for all the visceral arteries, with the exception of the abdominal aorta, in which an aneurism may arise from local increase of pressure.

2. The formation and further development of the aneurism is also favored by the narrowing of the arterial calibre, which is caused by the inflammatory swelling of its walls, and also by the contemporaneous formation of a thrombus (clot), this latter still further supporting and exciting the inflammation of the inner coat.

3. Whilst the causes above mentioned (and of these more particularly the continued presence of the palisade worms and the plugging of the smaller arteries by Thrombi) favor the growth of the worm-aneurism, the small size of the same, notwithstanding the years it has existed, is explained by the considerable hypertrophy of the muscular layer, by the tough fibrous capsule formed in many cases by the connective tissue of the mesentery, and by the adhesion of the intestines to the perpendicular and free-lying anterior mesenteric artery ; in particular this last-named circumstance does not allow of any very considerable shortening of the mesenteric artery, which would necessarily be accompanied by considerable dilation of the arterial tube.

4. The favorite seat of the worm-aneurism is the trunk of the anterior mesenteric artery, directly at its origin from the abdominal aorta. Most frequently that part of the arterial trunk is dilated from which the arteria ilea, cæcales, and colica inferior (arteria ileo-cæco-colica) arise, less frequently the arteria colica superior at its origin, and the arteries of the cæcum and colon in their course in the meso-cæcum and meso-colon. The verminous aneurism also occurs in the cœliac artery (Bauchschlagader), in the posterior mesenteric artery (Gekros-arterie), in the renal artery, and in the abdominal aorta. A horse is not unfrequently afflicted with several aneurisms of this kind at one and the same time. Thus in one case (described by Bollinger) there were six of these aneurisms affecting the abdominal aorta and its branches in the same horse. The verminous aneurism may occur from the sixth month of life onwards, and with increasing age; the number of horses free from such aneurisms becomes continually smaller.

5. The size of the aneurism varies between that of a pea and that of a man's head. The dilation is, as a rule, equal on all sides, the form being usually thumb-shaped or bottle-shaped, passing into that of a cone or long oval figure. This general configuration is principally due to the free and movable situation of the anterior mesenteric artery.

6. In contrast to aneurisms in man, the walls of the worm-aneurisms of the horse are almost without exception indurated. In addition to the mesenteric connective tissue, all the arterial coats, and especially the tunica media, generally take part in this induration. The hypertrophy of the media, which stands unique in respect of what is known of arterial disease, forms a compensatory action of the arterial wall, analogous to the muscular hypertrophy of the heart in valvular disease. This change in the media points to the fact that in the development of aneurism in man the early disturbance of the nutritive process in the tunica media is not a less essential factor than the degeneration of the tunica intima.

The changes in the intima are the least constant. They present all stages of progressive and retrogressive metamorphosis, from simple induration to ulceration and calcification. In the walls of the verminous aneurism one not unfrequently finds all the pathological changes exhibited by atheroma in man. Calcification is a common form of the retrograde process, and, in very rare cases, may pass on to the formation of true bone.

7. In addition to the palisade worms, one almost constantly finds a parietal thrombus contained in the aneurism. It covers the inner wall either partially or completely, being in the latter case perforated for arterial offshoots. This clot may occlude the artery, and it is not unfrequently continued into the arterial branches (peripherally) or into the aorta (centrally). Amongst the various changes that the clot undergoes, organization of its outermost layer and softening are the most frequent. The constant occurrence of this clot is due to the presence of the worms, to the inflammation, ulcerative and regressive affection of the intima, and to the dilatation of the arterial tube.

8. The palisade worms are seldom absent from aneurisms of the horse. Their not being present is merely an accidental circumstance. On the average, nine palisade worms go to a verminous aneurism, and eleven in the horse. The highest number of worms found in one horse reached 121. Not unfrequently, also, palisade worms, or their coverings in the form of larval skins, are found in the aneurismal walls. The immigration and emigration of the palisade worms out of the intestine into the aneurism, and the reverse, take place probably, as a rule, within the arterial circulation. The path of the worm does not appear to be always the same, inasmuch as they can also wander through the peritoneal cavity. The worms found in the aneurismal walls are probably mostly only strayed specimens.

9. From a comparative pathologico-anatomical point of view, the developmental history of the aneurysma verminosum proves that a circumscribed endo-arteritis can determine the formation of an aneurism.

10. Like the worm-aneurism itself, atheroma of the abdominal arteries arises from a circumscribed acute and subacute endo-arteritis. The histological changes in the secondary atheroma of horses are perfectly analogous to those of the spontaneous atheroma of man. Idiopathic atheroma, as seen in man, does not occur any more in the horse than in the other domestic animal. Atheroma in the horse is always secondary. To be sure, one observes an idiopathic chronic endo-arteritis in many abdominal arteries of the horse, which, however, never exhibits indications of atheromatous degeneration.

11. In consequence of its position the worm-aneurism of horses is not open to physical examination, and on that account cannot be diagnosed by physical signs; moreover, it offers no characteristic symptoms. Its termination by rupture is extremely rare, the aneurisms of the abdominal aorta being more disposed to rupture than those of the anterior mesenteric artery. Of eighteen cases of known perforation, fifteen opened into the peritoneal cavity, and three into the bowel. The dangerous symptoms of the worm-aneurism are exclusively due to embolism and thrombosis of the affected artery, arising from the parietal clot. The latter becomes especially dangerous through its increasing size and the softening which often accompanies it. The absorption and shrinking of this parietal clot, be it organized or not, is materially assisted by the high pressure to which it is exposed.

12. The very marked symptoms of vascular obstruction—the sero-hæmorrhagic intestinal infarct—in embolism and thrombosis of the mesenteric arteries are easily explained by paralysis of the muscular coat of the intestine, by the absence or paucity of valves in the portal vein, by the readiness with which meteorismus (or flatus) arises, especially in herbivora, and by the loose consistence of the intestinal walls or villi.

13. The occlusion of the intestinal arteries, especially that arising suddenly, always has for its result a partial or complete paralysis of the portion of bowel which they supply. The palsy of the intestine causes the forward movement of the intestinal contents to cease, a stoppage of the fæces, a hindrance to the discharge of fæces and gas, and also that exceedingly dangerous formation of gas (within the intestinal tract) which in the herbivora is so abnormal, both quantitatively and qualitatively.

14. In embolism and thrombosis of the mesenteric arteries, the symptoms during life are entirely identical with those observed in the so-called colic of horses, as has been determined by numerous observations. The partial paralysis of the bowel, which is brought on by the embolism and thrombosis of the mesenteric arteries, forms in great part the chief and leading feature of the series of symptoms known as the "colic" of horses. The palsy of the bowel which arises in this way may explain also the frequent ruptures of the digestive canal and the greater number of its changes in position. The latter are specially favored by the structure of the abdominal viscera in the horse.

15. The old changes which one finds in the peripheral branches of the anterior mesenteric artery, in the form of expired and partly absorbed embolic and thrombolic processes (pigmentation, arterial and venous thrombi), particularly in connection with those arteries which are seats of the aneurism, decisively prove that the large majority of colics resulting in recovery, so far as they do not depend upon known injuries, are caused by paralysis of the bowel from embolism and thrombosis. The sudden occurrence, course, and result of these kinds of colics also testify to their embolic origin.

16. The œdematous, inflammatory, and hæmorrhagic processes that one often finds described as the cause of death in colic, almost exclusively depend on thrombosis and embolism of the mesenteric arteries, the cases forming about 40 to 50 per cent. of all fatal colics.

17. The rapid course in fatal colics, as well as the preponderating symptoms of dyspnœa in cases of recovery, is finally due to the abnormal development of gas in the alimentary canal. In addition to the diminution of the respiratory surface by the lofty position of the diaphragm, a direct gas-poisoning (carbonic acid and sulphuretted hydrogen) probably contributes to the intensity of the symptoms and the rapid course by diffusion of the abnormally doveloped gas out of the intestinal canal into the blood.

18. The variety of the anatomical derangements caused by embolism and thrombosis of the intestinal arteries is faithfully mirrored by the variety of the clinical symptoms and the different degrees in the intensity and course of the colic.

19. Amongst every 100 horses afflicted with internal disease, 40 are ill with colic. Among any hundred deceased horses 40 have perished from colic, and among 100 colic patients 87 recover and 13 die. The figures prove that neither amongst the epizootic nor sporadic diseases of horses is there any other affection which occurs so frequently, or claims anything like so many victims. Like the frequency of the worm-aneurism, the amount of disease and mortality increases with advancing age. The etiology of the colic of horses finds in the thrombosis and embolism of the mesenteric arteries, with the consequent paralysis of the bowel, an all-sufficient explanation, whilst the causes of colic hitherto accepted were for the most part insufficient.

20. In a great number of cases the thrombus of the worm-aneurism is continued past the mouth of the anterior mesenteric artery, into the lumen of the aorta, and, as such, is the exclusive cause of the embolisms of the pelvic and crural arteries which bring about the intermittent hobblings (the author says "intermitterenden Hinken," not "Hahnentritten," the usual equivalent term for stringhalt). Considering the excessive frequency of the thrombus being continued into the aorta, it becomes highly probable that a great part of the diseases and lameness of the posterior extremities ("Huft und Kreuzlahme, unsichtbarer Spath, etc.," which may be rendered "sciatic and hip or spinal lameness, obscure spavin, etc.,") are due to occlusion of the arteries.

21. Owing to the fibrous thickening of the connective tissue of the root of the anterior mesenteric around the aneurism, and to the considerable size of the latter, disturbances of the innervation of the intestine, (as well as) hindrances to the passage of the chyle, and irregularities in the portal circulation may be created, which may well lie at the root of many chronic disturbances of digestion in horses.

22. Considering the great losses and heavy social disadvantages that are occasioned by the colic of horses to the horse-breeder, to agriculture, and to the general welfare, it is of the highest importance to discover means which should prevent the introduction of the embryos with the food, and, as a consequence, the migration of the palisade worms into the mesenteric arteries of the horse.

In calling your attention to this subject, old as it is, I hope I have not encroached too much on your valuable time.

If I have been able to cause one of you to ask yourself, can these statements made be sustained by post mortem examination—for I think we have been able to—then, with the interest of your profession at heart, I hope you will open the book of nature and determine the fact for yourselves.

### NOTE.

Three full page plates of illustrations of the strongylus armatus belong to this article. Through no fault of our own, they have been delayed or withheld, and will be furnished under separate cover, so that they can be inserted later.

We regret extremely that our readers should be subjected to this inconvenience, which has also seriously interfered with the completion of this number of the JOURNAL, and the publication of other valuable matter, which will, however, appear in the November number.

